# Exogenous Interleukin-37 Alleviates Hepatitis with Reduced Dendritic Cells and Induced Regulatory T Cells in Acute Murine Cytomegalovirus Infection

**DOI:** 10.1155/2023/1462048

**Published:** 2023-05-12

**Authors:** Yufei Ruan, Zhengwang Wen, Ke Chen, Jianan Xi, Bo Wu, Zhiyong Xu, Minzhi Jiang, Junling Zhang, Yiping Chen, Qi Liu

**Affiliations:** ^1^Department of Pediatric Infectious Disease, The Second Affiliated Hospital and Yuying Children's Hospital of Wenzhou Medical University, Wenzhou, Zhejiang Province 325027, China; ^2^Department of Emergency, Children's Hospital, Zhejiang University School of Medicine, Hangzhou 310003, China; ^3^School of Basic Medicine, Anhui Medical University, Hefei 230000, China

## Abstract

Human cytomegalovirus (HCMV) infection is globally distributed, and the liver is one of the major targeting organs. So far, the mechanisms for cell and organ damage have not fully been elucidated and the treatments for the infection are mainly at symptoms. IL-37 has shown a protective role in certain inflammatory diseases. In the present study, potential protective effect of exogenous IL-37 on murine cytomegalovirus- (MCMV-) infected hepatitis was evaluated through analyses of serum transaminases, the liver histopathology and cytokine expression, and functional state of dendritic cells (DCs) and regulatory T cells (Tregs). These analyses showed a significant decrease in serum transaminase levels and a lower Ishak histopathologic score at the early stage of MCMV-infected mice with exogenous IL-37 pretreatment. The frequencies of MHC-Ⅱ, CD40, CD80, and CD86 positive DCs in the liver and spleen were decreased significantly at 7 days postinfection (dpi) in MCMV-infected mice with IL-37 pretreatment when compared with those without the pretreatment, while the total number of DCs in the liver was reduced in IL-37-pretreated mice. The induction of Tregs in the spleen was enhanced at dpi 3 with IL-37 pretreatment in MCMV-infected mice. The mRNA expression levels of cytokines in the liver were decreased significantly (IL-1*β*, IL-6, IL-10, IL-4) or to some extent (TGF-*β* and TNF-*α*). The present study suggested that exogenous IL-37 can alleviate MCMV-infected hepatitis, likely through reduced DCs and induced Tregs with a weaker cytokine storm, demonstrating its potential value in clinical management for HCMV-infected hepatitis.

## 1. Introduction

Cytomegalovirus (CMV) is a beta-herpesvirus of the order Herpesviridae, with double-stranded DNAs [1]. Globally, seroprevalence of human cytomegalovirus (HCMV) is 40%–99% [2]. Studies have shown that HCMV is a common pathogen causing a variety of diseases in young children, and children younger than 1 year old are the main epidemic population of HCMV infection [3], of which congenital infection is often manifested as neurological sequelae such as hearing impairment, cognitive impairment, and microcephaly [4]. In addition, children with HCMV infection can present a wide range of cell and organ pathologies, such as pneumonia, infantile hepatitis syndrome, and infectious mononucleosis [3]. At present, no vaccine is available, and the treatment is mainly for symptoms, with antiviral compounds. Thus, studies on the mechanisms for HCMV-led cell and organ damage are still of pressing medical importance.

Prior studies have shown that CMV infection impairs the differentiation and maturation of myeloid cells, including CD34+ hemopoietic progenitor cells [5] and myeloid dendritic cells [6], the former is deemed as the reservoir of latent CMV. In contrast to plasmacytoid dendritic cells (pDCs), myeloid dendritic cells (mDCs, also called conventional DCs or cDCs) originate from common myeloid progenitor cells (CMP) that produce monocytes, dendritic cells, and macrophages. As sentinel cells, mDCs can activate naive T cells and initiate adaptive immune responses. Recently, Xue et al. [7] report that islet recipient mice transfused with immature DCs present prolonged islet survival with increased regulatory T cells (Tregs) and reduced Th17 cells in the spleen and draining lymph nodes. The mDCs express abundant costimulatory molecules, such as MHC Class Ⅰ and Ⅱ molecules, CD40, CD80, and CD86 which are involved in activating naive T cells. Various pathogen-derived microbial molecules combined with toll-like receptors can trigger mDCs maturation [8]. Matured mDCs express high levels of T-cell costimulatory molecules [9]. Previous studies have shown that in the case of HIV infection, mDCs may provide CD4^+^ T cells with a signal to inhibit viral gene expression and promote the establishment and maintenance of viral latency [10].

IL-37, as a new inhibitory cytokine in inflammatory response and innate immunity, is encoded by the IL-1 family gene cluster on human chromosome 2 and consists of six exons. It expresses five different transcripts (a–e), among which IL-37b is the most complete (including five of the six exons) and abundant [11–13]. However, wild-type mice do not have the IL-37 gene, so many studies have used recombinant human IL-37 for intervention or transgenic mice in experiments [14]. Endogenous IL-37 can enter the nucleus in a Smad3-dependent manner to inhibit the transcription of proinflammatory genes [15]. Exogenous IL-37 binds to IL-18 and co-receptor IL-1R8 to form a triplet complex to transmit anti-inflammatory signals. Studies have found that IL-37 can regulate the differentiation and maturation of DCs, which modulates the activation of naive T cells and the proliferation of effector T cells [16–18]. IL-37 may play a protective role in a variety of diseases [19], mainly inflammatory diseases and autoimmune diseases [20–22], demonstrating its potential for clinical management in these diseases.

The present study aimed to test a hypothesis on a protective role of IL-37 in CMV-induced hepatitis in a mouse model of acute murine CMV (MCMV) infection. With IL-37 pretreatment, the induction and maturation of mDCs in the liver and spleen of the MCMV-infected mice were analyzed, along with examination of liver function and histopathological changes. The amount and function state of Tregs, along with the main inflammatory cytokines, were also analyzed in the mouse model. The current study demonstrated that exogenous IL-37 might have played a protective role in MCMV-infected hepatitis, with reduced DCs and induced Tregs in MCMV-infected mice.

## 2. Materials and Methods

### 2.1. Cell and Virus

Primary murine embryonic fibroblasts (MEF) were prepared from embryos on day 14. MCMV (Smith strain) was supplied by the Institute of Clinical Virology of Anhui Medical University, propagated according to the protocol described by Brizić et al. [23], and cocultured with primary MEF. The viral titer was determined at 6.5 × 10^6^ plaque-forming units (PFU)/ml.

### 2.2. Animal and Experimental Design

Male, wild-type C57BL/6J mice aged 4–6 weeks and weighted 16–18 g, were obtained from the Animal Center of the Chinese Academy of Sciences (Shanghai, China) and housed at the Laboratory Animal Center under the standard settings (23°C ± 2°C, 60% ± 10% humidity, and 12 hr light/dark cycle). All animal experiments were approved by the Experimental Animal Ethics Committee of Wenzhou Medical University and carried out in accordance with the guidelines for the care and use of laboratory animals of the National Institutes of Health. Mice were randomly divided into four groups: phosphate-buffered saline (PBS) pretreatment, IL-37 pretreatment, PBS plus MCMV infection, and IL-37 plus MCMV infection (*n* = 3 − 5 mice in each group). PBS and IL-37 were given one day before MCMV infection that was established by intraperitoneal injection of 200 *μ*l MCMV suspension at 1.0 × 10^6^ PFU. Treatment with 1 ng (200 *μ*l) of recombinant human IL-37 or equal volume of PBS was applied to corresponding groups of mice. Mock infection was achieved by intraperitoneal injection of equal volume of MEF suspension. At 3, 7, and 14 days postinfection (dpi), under the complete anesthesia of pentobarbital sodium, mouse blood was taken by cardiac puncture and tissue specimens of the liver and spleen were taken for histological examination and immunological analysis.

### 2.3. Assessment of Liver Function and Histology

The whole blood was centrifuged at 3,000 r/min for 20 min to obtain the serum, and then serum alanine/aspartate aminotransferases (ALT and AST) were measured in an automatic biochemical analyzer (Hitachi 7000, Japan). The liver tissues were fixed with 4% paraformaldehyde, embedded in paraffin, and sectioned for histological assessment with hematoxylin–eosin (HE) staining. Under the inverted fluorescent microscope (Olympus, Japan), three randomly selected nonoverlapping fields (×200 magnification) were used for pathological scoring, according to the Ishak scoring system [24, 25], a commonly-adopted standard for histopathology of clinical viral hepatitis.

### 2.4. Isolation of Liver and Spleen Mononuclear Cells

Under anesthesia with pentobarbital sodium, the liver was perfused with sterile Hank's balanced salt solution (HBSS, pH 7.4, Thermo, USA) via the portal vein. When the liver turned pale, it was resected along with the spleen. The liver and spleen tissues were ground through a 70 *µ*m cell strainer to obtain single cell suspensions and digested with 0.2 mg/ml collagenase Ⅳ (Sigma-Aldrich, USA) at 37°C for 30 min [26]. The suspensions of liver and spleen homogenates were subjected to the density gradient centrifugation with 40% and 70% Percoll solutions (Cytiva, USA) at 800 g for 30 min, and then the white cell enrichment layer, namely mononuclear cells, was collected and resuspended in PBS following the removal of red blood cells with ACK lysis buffer (Beyotime, China) [27, 28].

### 2.5. Cell Staining and Flow Cytometry

All cell suspension samples were adjusted at 10^6^ cells per 50 *μ*l before running the flow cytometry. Cells were then incubated for 10 min at 4°C with anti-mouse CD16/32 (Biolegend, USA) for blocking Fc receptors. Surface molecules of DCs were stained for 30 min at 4°C with the following antibodies: anti-mouse CD11c Percp-cyanine 5.5 (eBioscience, USA), anti-mouse lineage brilliant violet 421, anti-mouse B220-APC-Cy7, anti-mouse CD40-APC, anti-mouse CD80-PE, anti-mouse CD86-FITC, and anti-mouse MHC-Ⅱ PE-cyanine 7 (Biolegend, USA). Treg cells were stained with surface markers: anti-mouse CD4-FITC (Biolegend, USA) and anti-mouse CD25-APC (eBioscience, USA). The intranuclear Foxp3 of Treg cells was stained with anti-mouse Foxp3-PE (eBioscience, USA) following cell fixation and permeabilization with the transcription factor buffer set (BioLegend, USA). Cellular fluorescence was assessed with FACS Canto II (BD Biosciences, USA) and data were analyzed with FlowJo software (version 10.2, TreeStar, USA) [29].

### 2.6. RNA Extraction and Quantitative RT-PCR

The liver tissues were lysed with lysate buffer RL, and the RNA was purified with universal RNA Extraction Kit (Takara, Japan). Quantitative RT-PCR was performed with the TB Green Premix Ex Taq Ⅱ (Takara, Japan) on a real time PCR system (Roche, Lightcycler 480, CH). Mouse IL-1*β*, IL-4, IL-6, IL-10, TGF-*β*, TNF-*α*, and GAPDH primers were purchased from the General Biol Biotechnology Company (Chuzhou, Anhui, China). The *ΔΔ*CT method with GAPDH as internal reference gene was used for the analysis of relative expression levels of the target genes. Primer sequences are shown in Table 1.

### 2.7. Statistical Analysis

Statistical tests were performed with SPSS 23.0 (SPSS Software, USA) and GraphPad Prism 8.0.2. (GraphPad Software, USA). The quantitative data were presented by mean ± standard deviation. Appropriate statistical methods were selected based upon the data distribution and characteristics: for comparisons between two groups, Student's *t*-test was used when data were or approximated to normal distribution, or Wilcoxon paired test was used for skewness distribution data. One-way analysis of variance (ANOVA) was used for comparisons across multiple groups, and then LSD or Bonferroni (homogeneity of variance) or Dunnett's T3 test (heterogeneity of variance) were used for multiple test correction. The repeated measurements on the same animal in RT-qPCR assays were analyzed using the spline regression method with duplication correlation adjustment to compare general differences between two groups over time series. *P* < 0.05 was regarded as statistically significant.

## 3. Results

### 3.1. IL-37 Alleviates MCMV-Induced Acute Liver Damage

**Figure 1:**
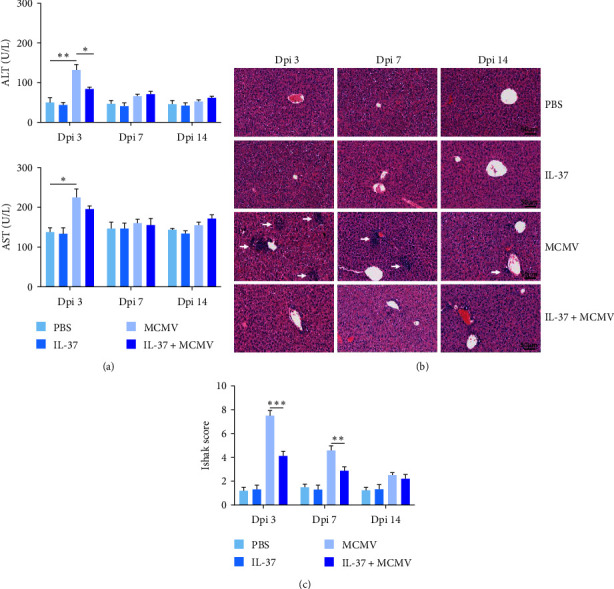
Protective effect of exogenous IL-37 on MCMV-induced hepatitis in mice (*n* = 3 − 5 mice in each group): (a) quantitative analysis of serum ALT and AST at dpi 3, 7, and 14 ( ^*∗*^*P* < 0.05,  ^*∗∗*^*P* < 0.01,  ^*∗∗∗*^*P* < 0.001); (b) representative images of liver sections rom mice of the four groups at dpi 3, 7, and 14, scale = 50 *μ*m. (c) Quantitative analysis of Ishak score ( ^*∗*^*P* < 0.05,  ^*∗∗*^*P* < 0.01,  ^*∗∗∗*^*P* < 0.001) of liver in experimental groups on dpi 3, 7, and 14.

Without IL-37 pretreatment, MCMV-infected mice at dpi 3 were presented with significantly elevated serum ALT and AST levels when compared with uninfected mice (MCMV vs. PBS groups, *P* < 0.01–0.05, Figure 1(a)), which, along with histopathological changes described below, confirms MCMV-induced acute hepatitis. Interestingly, at dpi 3, MCMV-infected mice with IL-37 pretreatment had significantly reduced serum ALT levels when compared with those MCMV-infected mice without IL-37 pretreatment (MCMV + IL-37 vs. MCMV groups, *P* < 0.05). The serum AST levels were also decreased to a certain extent (*P* = 0.222) in these mice. Reduction of serum ALT level by IL-37 pretreatment observed at dpi 3 was not evident at dpi 7 and 14 in MCMV-infected mice (Figure 1(a)). No significant changes in those liver enzyme levels were observed between PBS and IL-37 groups in uninfected mice at dpi 3, 7, and 14.

Without IL-37 pretreatment, liver histopathological changes were most significant at dpi 3 in MCMV-infected mice, with disordered arrangement of hepatic cells, focal necrosis, and inflammatory infiltration (Figure 1(b)). These pathological changes nearly recovered at dpi 14. In contrast, IL-37 pretreatment significantly improved liver pathology and reduced inflammatory cell infiltration in MCMV-infected mice. No liver histological changes were observed in uninfected mice, where hepatocytes were intact and arranged radially around the central vein (Figure 1(b)). Quantitatively, assessment with the Ishak scoring system indicated that IL-37 had a protective effect on the liver pathological changes in MCMV-infected mice. Specifically, MCMV-infected mice with IL-37 pretreatment had significantly lower Ishak scores than those without IL-37 pretreatment at dpi 3 (4.1 ± 1.0 vs. 7.4 ± 1.3, *P* < 0.001) and dpi 7 (2.9 ± 0.9 vs. 4.6 ± 1.1, *P* < 0.01, Figure 1(c)).

### 3.2. IL-37 Inhibits MCMV-Induced Accumulation and Maturation of DCs in Mouse Liver

**Figure 2:**
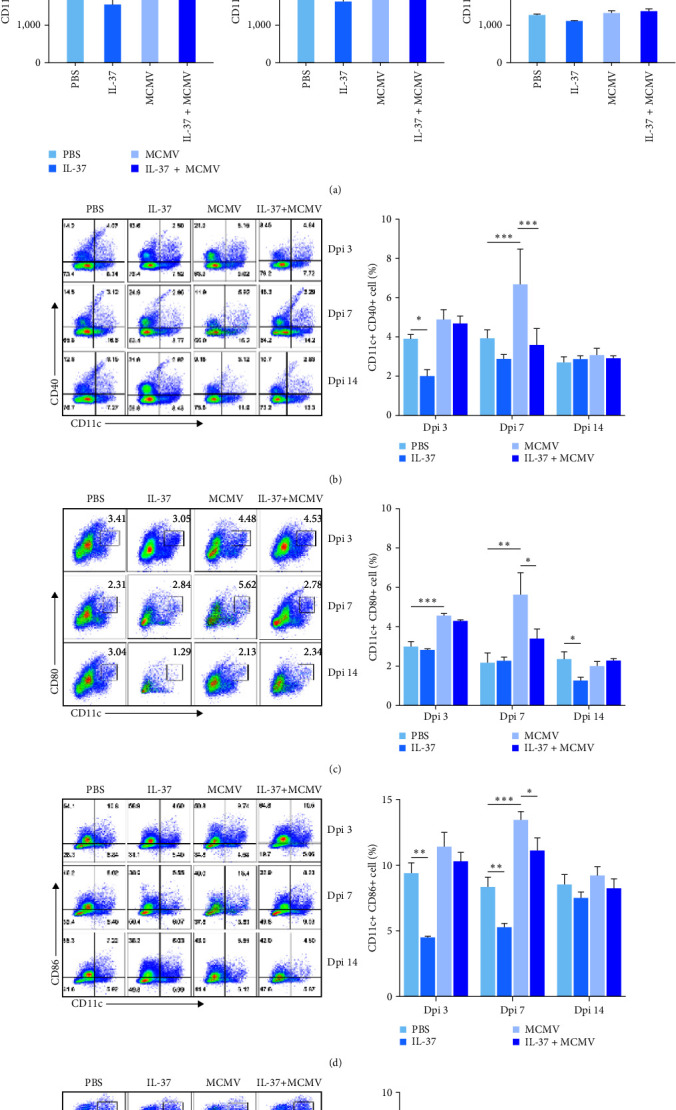
Effects of IL-37 on the accumulation and maturation of DCs in the liver of MCMV-infected mice at the early stage of viral hepatitis (*n* = 3 − 5 mice in each group): (a) distribution and quantitative analysis of CD11^+^ cells with the area under curve (AUC) at dpi 3, 7, and 14; (b–e) the frequencies of MHC-Ⅱ-, CD40-, CD80-, and CD86-positive DCs at dpi 3, 7, and 14 and quantitative analysis of DC subsets in flow cytometry.  ^*∗*^*P* < 0.05,  ^*∗∗*^*P* < 0.01,  ^*∗∗∗*^*P* < 0.001.

To explore the role of IL-37 in the regulation of DC response, total number of DCs in the liver, a major target organ in MCMV infection, was measured. It was found that the total number of liver DCs was increased significantly in MCMV-infected mice when compared with that in uninfected mice at dpi 3 (*P* < 0.001, Figure 2(a)) and so was at dpi 7 between MCMV and PBS groups (*P* < 0.05), which reflects active immune response against MCMV infection. At dpi 14, the total number of DCs in the liver of MCMV-infected mice was returned to the baseline of uninfected mice. In MCMV-infected mice, IL-37-pretreatment led to a downward trend in the number of liver DCs when compared with PBS-pretreatment at dpi 3 and 7. In uninfected mice, no significant difference in the total number of liver DCs was found between PBS- and IL-37-pretreatments at dpi 3, 7, and 14, indicating that IL-37 had no significant effect on the total number of liver DCs in the healthy mice (Figure 2(a)).

With regards to pDC and cDC subsets, while there was no effect of IL-37 pretreatment on frequencies of pDC in both uninfected and MCMV-infected mice (Figure S1a), the frequencies of cDCs, represented by overall intensity of MHC-II, CD40, CD80, and CD86 (Figure 2(b)2(e)), were significantly increased in MCMV-infected mice when compared with uninfected mice (MCMV vs. PBS groups, all four molecules *P* < 0.001–0.01 at dpi 7; two of the four *P* < 0.001–0.01 (MHC-Ⅱ and CD80) at dpi 3), but these increases were significantly reduced with IL-37 pretreatment (all four *P* < 0.001–0.05 at dpi 7; one of the four *P* < 0.01 (MHC-Ⅱ at dpi 3). The reduction of the surface-marker positive cDCs in the liver of MCMV-infected mice by IL-37 pretreatment suggests that the accumulation and maturation of cDCs might have been inhibited by IL-37.

### 3.3. IL-37 Inhibits MCMV-Induced Early Accumulation and Maturation of DCs in Mouse Spleen

**Figure 3:**
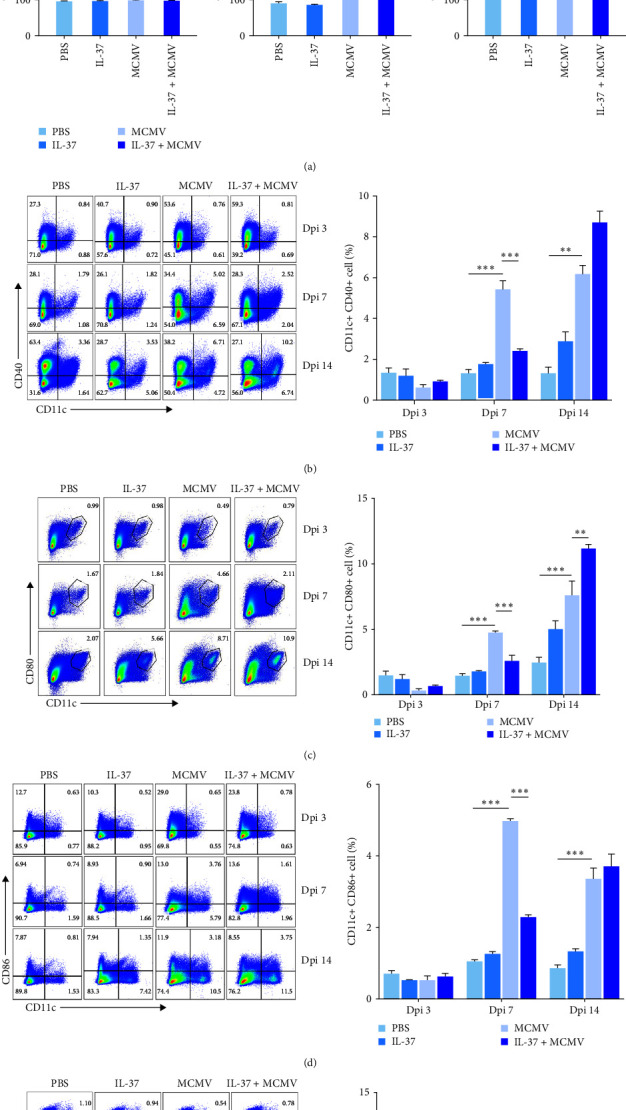
Effects of IL-37 on MCMV-induced accumulation and maturation of DCs in the spleen of MCMV-infected mice (*n* = 3 − 5 mice in each group): (a) distribution and frequency of CD11^+^ cells at dpi 3, 7, and 14; (b–e) frequencies of MHC-Ⅱ-, CD40-, CD80-, and CD86-positive DCs at dpi 3, 7, and 14 and quantitative analysis of DC subsets in flow cytometry.  ^*∗*^*P* < 0.05,  ^*∗∗*^*P* < 0.01,  ^*∗∗∗*^*P* < 0.001.

Flow cytometric analysis of DCs in the spleen was conducted, as the spleen is the largest peripheral immune organ. It was found that the total number of splenic DCs in MCMV-infected mice was increased significantly at dpi 7 and 14 when compared with that in uninfected mice (MCMV vs. PBS groups, *P* < 0.05–0.01, Figure 3(a)).

Similarly to the liver pDC, there was no significant difference in the number of splenic pDC subsets between MCMV-infected mice with and without IL-37 pretreatment at dpi 3, 7, and 14 (Figure S1b). Intriguingly, the same pattern of cDC frequency changes as identified in the liver was also seen in the spleen at dpi 7, that is, the frequencies of the surface marker positive cDCs in MCMV-infected mice were increased significantly when compared with those in uninfected mice (MCMV vs. PBS groups, all four molecules *P* < 0.001, Figure 3), Notably, these increases were significantly reduced in MCMV-infected mice with IL-37 pretreatment (all *P* < 0.001). Unlike in the liver, frequencies of the surface marker positive cDCs stayed at higher levels at dpi 14 in MCMV-infected mice than those in uninfected mice (MCMV vs. PBS groups, all *P* < 0.001–0.01). These increased frequencies of splenic cDCs in MCMV-infected mice were induced to even higher levels with IL-37 pretreatment, which was evidenced by significantly increased intensities of MHC-Ⅱ (11.2 ± 0.3% vs. 7.2 ± 1.3%, *P* < 0.05) and CD80 (11.2 ± 0.424% vs. 7.6 ± 1.57%, *P* < 0.01) and an upward trended intensity of CD40 and CD86 (Figure 3).

### 3.4. IL-37 Promotes MCMV-Induced Early Accumulation and Activation of Tregs in Mouse Spleen and Liver

**Figure 4:**
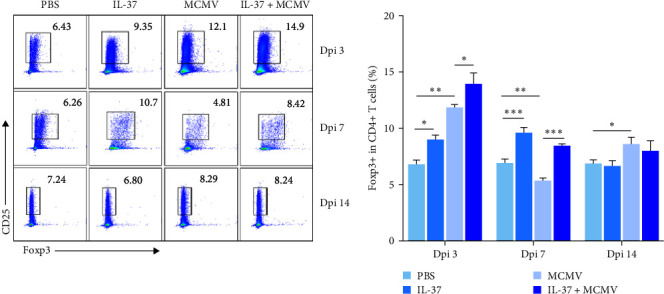
Effects of IL-37 on the induction and activation of Tregs in the spleen of MCMV-infected mice (*n* = 3 − 5 mice each group). Representative diagrams of flow cytometry and quantitative analysis of CD4+ CD25+ Foxp3+ Tregs in the spleen of mice at dpi 3, 7, and 14.  ^*∗*^*P* < 0.05,  ^*∗∗*^*P* < 0.01,  ^*∗∗∗*^*P* < 0.001.

To explore the effect of IL-37 on Treg response in MCMV infection, the frequency of Tregs in mouse spleen and liver was analyzed with the Treg classic markers (CD25 and Foxp3) in the flow cytometry. Frequencies of Tregs were compared between MCMV-infected mice with PBS or IL-37 pretreatment to determine whether IL-37 could promote an induction of Tregs in the spleen and liver. As shown in Figure 4, frequencies of splenic Tregs in MCMV-infected mice were significantly higher than that in uninfected mice at dpi 3 and 14 (MCMV vs. PBS groups, *P* < 0.01–0.05). More importantly, frequencies of splenic Tregs were increased significantly with IL-37-pretreatment when compared with PBS-pretreatment in both MCMV-infected and uninfected mice at dpi 3 (13.9 ± 1.3% vs. 11.9 ± 0.3%, *P* < 0.05) and dpi 7 (8.4 ± 0.3% vs. 5.3 ± 0.5%, *P* < 0.001), implicating that IL-37 might have promoted the induction of Tregs in the mouse spleen, regardless of MCMV infection. Of note, such an induction of Tregs promoted by IL-37 pretreatment was not observed at dpi 14 (*P* > 0.05).

In the liver, there was no significant difference in the frequencies of liver Treg between MCMV-infected and uninfected groups (*P* > 0.05). Notably, IL-37-pretreatment led to significantly higher frequencies of liver CD25+ Foxp3+ Tregs in both MCMV-infected and uninfected mice at dpi 3 when compared with those without IL-37-pretreatment MCMV infection: 12.7 ± 2.3% vs. 6.2 ± 2.2%, *P* < 0.01 and uninfected: 12.4 ± 3.1% vs. 6.8 ± 0.7%, *P* < 0.01. The fact that such a significant change in liver Treg was seen at dpi 3 but not at dpi 7 and 14 might indicate that IL-37 could have promoted the proliferation of the liver Tregs, predominantly at the early stage in both MCMV-infected and healthy mice.

### 3.5. IL-37 Reduces IL-1*β*, IL-6, TGF-*β*, and IL-10 mRNA Expression in the Liver

**Figure 5:**
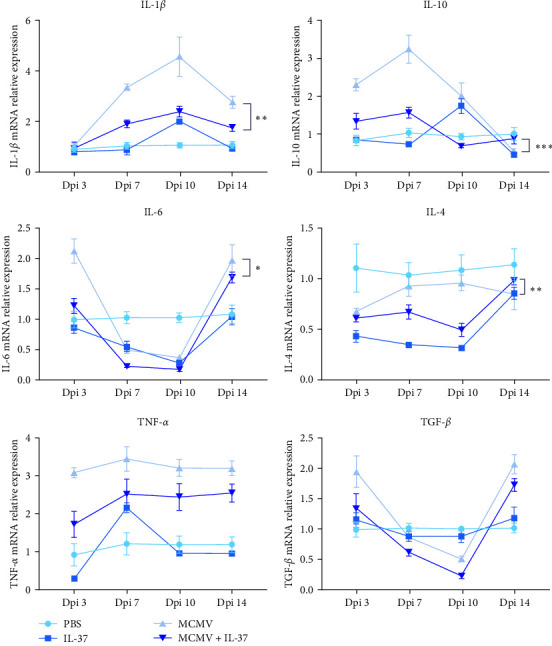
Effects of IL-37 on cytokine expression in the liver of MCMV-infected mice (*n* = 3 − 5 mice in each group). Each colored dot is for the mean value of a group and the vertical lines represent the standard error in a group of mice (*n* = 3).  ^*∗*^*P* < 0.05,  ^*∗∗*^*P* < 0.01,  ^*∗∗∗*^*P* < 0.001.

Cytokines play an indispensable role in the immune response to MCMV infection. To robustly detect general differences between IL-37 pretreatment and MCMV-only curves, one more timepoint dpi 10 was introduced in cytokine qPCR assays. In the MCMV infection, mRNA expression levels of IL-1*β* (*P* < 0.01), IL-4 (*P* < 0.01), IL-6 (*P* < 0.05), IL-10 (*P* < 0.001), and TGF-*β* over the four timepoints were significantly reduced in IL-37-pretreated mice when compared with PBS-pretreated mice, whereas the mRNA levels of TNF-*α* and TGF-*β* were in downward trend from MCMV-only to MCMV+IL-37 groups (*P* = 0.31 and 0.29, respectively) (Figure 5).

## 4. Discussion

Human and murine CMVs have homologous genes, and MCMV mouse models can simulate multiple disease models except for congenital cytomegalovirus infection [30]. Different mouse strains have different responses to MCMV infection. Here we wanted to point out the uniqueness of the C57BL/6J mouse strain for CMV-induced acute hepatitis model. With the genetic locus *CMV1* encoding NK cell receptor Ly49H, C57BL/6J mice show highly active natural killer cells that render substantial resistance to MCMV infection [23]. Such a genetic feature may explain a relatively shorter course of acute MCMV-infected hepatitis observed in the present study, where the experimental assays for the protective and immunoregulatory effects of IL-37 were focused on the early stage of the MCMV infection, that is, at 3 and 7 days postinfection.

The present study observed a protective effect of IL-37 on MCMV infected hepatitis, with significantly reduced serum transaminase levels in MCMV-infected mice by IL-37 pretreatment. This was statistically evident with ALT level and with AST reduction to some extent. The liver histopathology and Ishak scoring system further confirmed reduced liver inflammation in IL-37 pretreated mice. Zhao et al. [31] showed that IL-37 can reduce the proinflammatory cytokines and chemokines produced by a variety of hepatocytes and Kupffer cells, such as TNF-*α*, MIP-2, and KC, and prevent from the subsequent recruitment of neutrophils [32]. Moreover, Li et al. [33] demonstrated that when overexpressed, IL-37 can reduce the Th1 response by inhibiting the production of IL-12 by macrophage. It has also been proposed that IL-37 can reduce Con-A-induced hepatitis by inhibiting the activity of DCs, blocking the biological effect of CD4+ T cells, increasing the proportion of Tregs and enhancing their function [33]. Alongside these prior studies, the present study added evidence for an IL-37-associated protective effect on MCMV-infected hepatitis.

To explore the protective mechanisms of IL-37 on MCMV-infected hepatitis, the current study focused on the changes in certain key immune cells, such as DCs and Tregs. It was found that with IL-37 pretreatment, MHC-Ⅱ, CD40, CD80, and CD86 positive DCs in the liver and spleen of MCMV-infected mice were significantly decreased from the basis of MCMV alone induced elevation, most typically at the early timepoint dpi 7 in the comparison to mice without the pretreatment, suggesting IL-37-associated inhibition on the accumulation and maturation of DCs that led to tolerance phenotypes of DCs in the liver and spleen of MCMV-infected mice. Lunding et al. [18] showed that exogenous recombinant human IL-37 can reduce the size and area of atherosclerotic plaque in ApoE-deficient mice, indicating that IL-37 inhibits the differentiation and maturation of DCs and reduces the polarization of Th1 and Th17 cells, and thus have played an anti-inflammatory role. Of note, IL-37 had no significant effect on the frequency of pDCs subset in the present study, indicating a predominant effect of IL-37 pretreatment on cDC subsets [34]. While a similar pattern in changes of DC frequencies was observed in both liver and spleen of MCMV-infected mice, the splenic DCs were significantly activated at dpi 14 in uninfected mice as well, with upregulation of those surface molecules including pDC subset to a certain extent. This finding has not yet been reported in the literature and may reflect the discrepancies between the target organ liver and the immune organ spleen in response to MCMV and/or IL-37 pretreatment. Future experiments will be required to clarify its significance in the MCMV infection.

It has been shown [17] that DCs of IL-37 transgenic mice exhibit the characteristics of tolerance-derived phenotype, including decreased ability to stimulate the proliferation of naive and antigen-specific effector T cells, which results in downregulation of Th1, Th2, and Th17 and upregulation of Tregs functions, so as to play a role in controlling the excessive immune response. Recently, Popovic et al. [35] demonstrate that liver Treg cells are strongly induced in mice-infected with MCMV. The present study showed that IL-37 significantly promoted the accumulation and activation of Tregs in the liver of both uninfected and MCMV-infected mice at dpi 3. Such an effect was observed also in the spleen of MCMV-infected mice at dpi 3 and 7; however, it was not detected in the liver at dpi 7 and 14. Such an early effect might be due to the fading of IL-37 pretreatment, or the acute infection was rapidly controlled by the host immunity in the liver. Amo-Aparicio et al. [36] have found in the spinal cord injury model that the spinal cord injury of experimental mice was significantly reduced on the 3rd and 7th day by injecting exogenous IL-37 into the spinal cord. These studies suggest that IL-37 plays a major role in the early stage of the disease.

CMV infection can stimulate a wide range of host–immune responses and initiate the differentiation and activation of various immune cells [37]. Among them, the host cytokine microenvironment and the activity of key transcription factors are important determinants of the differentiation and maturation of myeloid cells, including DCs [38]. In addition, cytokines have multiple functions on the cells of the immune system, because some cytokines are produced by the cells of the immune system, while some cytokines also influence the production of other cytokines, and that all of this makes a complex cytokine network that constitutes complex immune mechanisms [39], of which the levels of anti-inflammatory and proinflammatory cytokines can be decisive for the development of inflammatory diseases. The current study showed that IL-37 significantly or to some extent decreased the expression levels of both proinflammatory (IL-1*β*, IL-6, TNF-*α*) and anti-inflammatory cytokines (IL-10, IL-4, TGF-*β*) and thus reduced cytokine storm-related liver damage in the MCMV infection. These findings are consistent with prior studies on IL-37 in a variety of inflammatory diseases [31, 32, 40, 41].

In conclusion, the present study suggested that exogenous IL-37 can inhibit the accumulation and maturation of cDCs in the liver and spleen of mice at the early stage of acute MCMV infection, and it also promoted the induction of Tregs in the spleen and suppressed the cytokine storm, all leading to the overall control of the excessive immune response and thus alleviated MCMV-infected hepatitis. It should be noted that cDC has been impacted apparently in the liver by both MCMV and IL-37 treatment, while Treg cells are primarily affected in the spleen by the IL-37 pretreatment. The findings from the current study demonstrate the potential value of exogenous IL-37 in clinical management for HCMV-infected hepatitis.

## Figures and Tables

**Table 1:** Primer sequences used for quantitative RT-PCR.

Mouse gene	Forward primer (5′-3′)	Reverse primer (5′-3′)
GAPDH	GAGAGTGTTTCCTCGTCCCG	ACTGTGCCGTTGAATTTGCC
IL-*β*	TCATCTTTGAAGAAGAGCCCA	TTGAGGTGGAGAGCTTTCAG
IL-4	GGTCTCAACCCCCAGCTAGT	GCCGATGATCTCTCTCAAGTGAT
IL-6	CTGCAAGAGACTTCCATCCAG	AGTGGTATAGACAGGTCTGTTGG
IL-10	TGCAGTGTGTATTGAGTCTGCT	TGTCCCCAATGGAAACAGCTT
TGF-*β*	GCAACAATTCCTGGCGTTACCT	GAAAGCCCTGTATTCCGTCTCC
TNF-*α*	GAACTGGCAGAAGAGGCACT	GGCCATTTGGGAACTTCTCATC

## Data Availability

The data that supports the findings of this study are available in the supplementary material of this article.

## Supplementary Materials

Supplementary Materials Figure S1(a, b): The proportion of B220 + pDC subset at dpi 3, 7, and 14 measured by flow cytometry and statistical analysis in the liver (a) and spleen (b).
