# Supplementary material for: Exogenous Interleukin-37 Alleviates Hepatitis with Reduced Dendritic Cells and Induced Regulatory T Cells in Acute Murine Cytomegalovirus Infection
Source: J Immunol Res. 2023 May 12;2023:1462048. doi: 10.1155/2023/1462048 (PMC10198762; doi:10.1155/2023/1462048)
Supplement: Supplementary Materials — Figure S1(a, b): The proportion of B220 + pDC subset at dpi 3, 7, and 14 measured by flow cytometry and statistical analysis in the liver (a) and spleen (b). [file 1462048.f1.docx]

**Supporting Information**


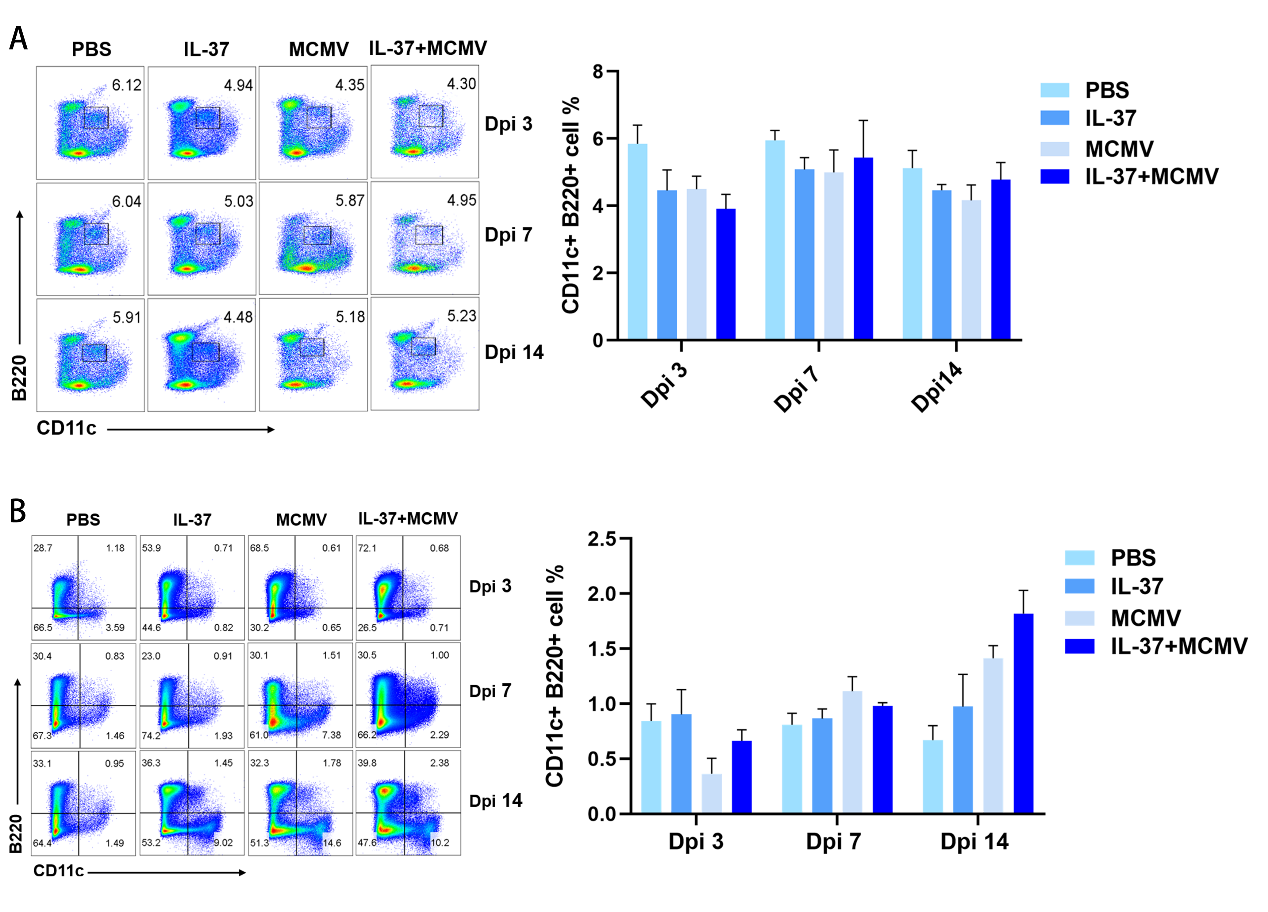


**Fig.S1**. The proportion of B220+pDC subset at dpi 3, 7 and 14 measured by flow cytometry and statistical analysis in the liver (A) and spleen (B). **P* < 0.05, ***P* < 0.01, ****P* < 0.001.
